# The First‐Hand Needs of Informal Caregivers of People Living with Dementia, in Residential Care Settings: A Scoping Review

**DOI:** 10.1002/alz70858_098551

**Published:** 2025-12-25

**Authors:** Chloe Moody

**Affiliations:** ^1^ University of Bath, Bath, Somerset, United Kingdom

## Abstract

**Background:**

Dementia is a terminal condition often requiring palliative care delivered in residential care settings. While informal caregivers (ICGs) are pivotal in care‐based decision‐making, they have higher rates of physical and mental illness than ICGs of people with other terminal conditions. Identifying the needs of ICGs of people living with dementia (PLwD) is essential, to mitigate these risks and develop effective support systems.

**Method:**

Following the JBI methodology for scoping reviews, electronic databases (APA PsychNet, the Cochrane Database of Systematic Reviews, PubMed and Web of Science) were searched in September 2024, with no publication date limitations. Thematic synthesis was conducted on the findings of eligible peer‐reviewed and grey literature, written in English, and reported in accordance with the PRISMA‐ScR checklist.

**Result:**

Forty‐six articles were included. There were three overarching themes: “knowledge and understanding of dementia”, “engagement in care‐based decisions”, and “coping mechanisms and support for own wellbeing”. Sub‐themes presented an interplay between these, demonstrating the importance of understanding dementia, the significance of such knowledge for ICGs to maintain their own wellbeing, subsequently influencing their engagement in care‐based decision making.

**Conclusion:**

Care settings must work towards compassionate and timely support for ICGs, including a stable point of contact throughout admission and should use lay language. Future studies should take a longitudinal approach to understand the evolving role of ICGs, with particular attention to cultural and ethnic needs.

